# An engineering-informed voting ensemble framework for rapid seismic risk screening of reinforced concrete structures

**DOI:** 10.1038/s41598-026-52799-8

**Published:** 2026-05-12

**Authors:** Özgür Pürsünlü, Abdulkadir Çevik, Ahmet Emin Kurtoğlu, Derya Bakbak

**Affiliations:** 1 Department of Civil Engineering, Gaziantep University, Gaziantep, Turkey; 2 Department of Civil Engineering, Iğdır University, Iğdır, Turkey; 3 Grand National Assembly of Türkiye (TBMM), Ankara, Turkey

**Keywords:** Machine learning, Earthquake damage assessment, Reinforced concrete buildings, Post-earthquake screening, Seismic risk, Decision-support systems, Engineering, Natural hazards, Solid Earth sciences

## Abstract

Rapid evaluation of existing building stocks is essential for reducing earthquake-related disaster risk, particularly in seismically vulnerable cities such as Gaziantep, Türkiye. However, commonly used assessment approaches are either time-consuming or rely on simplified linear models that fail to capture the complex relationship between seismic demand and building characteristics. This study proposes a safety-first ensemble data-driven screening framework for rapid post-earthquake building damage assessment, combining XGBoost, LightGBM, and CatBoost classifiers via soft voting. The framework is trained and tested using a large post-earthquake dataset from the Nurdağı and Islahiye districts, obtained from the Ministry of Environment, Urbanization and Climate Change, which includes georeferenced building attributes and officially assigned damage states without secondary reinterpretation. Spatially varying seismic demand is represented using interpolated peak ground acceleration values for DD-1 and DD-2 hazard levels, while V_S30_ parameters characterize local site conditions. Design spectral acceleration values are derived in accordance with the seismic codes applicable at the time of construction. To enhance physical interpretability, the model incorporates engineering-informed feature engineering, including interaction terms linking building height, construction period, and seismic demand. Model optimization prioritizes life safety by emphasizing recall over overall accuracy. The proposed framework achieves a recall of 88.9% on independent test data and zero false-negative predictions for high-rise reinforced-concrete buildings (≥ 6 storeys). Additional robustness analyses demonstrate stable performance under moderate data uncertainty. The results indicate that the proposed approach provides a reliable, interpretable, and safety-oriented decision-support tool for rapid seismic risk screening of reinforced concrete building inventories following major earthquakes.

## Introduction

On February 6, 2023, a catastrophic earthquake doublet occurred in the southeastern part of Türkiye, a Mw 7.7 (7.8) Pazarcık earthquake at 04:17 local time and a Mw 7.6 Elbistan earthquake at 13:24 local time, which caused extensive damage in 11 provinces, including Gaziantep^1–5^. These disasters, known collectively as the Kahramanmaraş earthquake sequence (KMES), caused more than 50,000 deaths, over 115,000 injuries, and economic damage of 34.2 billion to 110 billion, accounting for 4-11.6% of the GDP of Türkiye^6–8^. Gaziantep, which is about 53 km away from the Elbistan epicenter and 120 km away from the Pazarcık epicenter, was also severely affected, with 4,060 buildings destroyed and 14,047 buildings severely damaged^9^. This disaster underlined the urgent need for fast and reliable seismic assessment methods, since the manual assessment of the 14,047 severely damaged buildings would be overwhelmingly time-consuming for the emergency teams.

In addition to the scale of damage, the availability of a comprehensive, building-level post-earthquake dataset in the Gaziantep region provides a unique opportunity to investigate the relationship between observed damage and site-specific seismic demand^10^. After the 2023 earthquake series, damage data compiled by the Ministry of Environment, Urbanization, and Climate Change in the Nurdagi and Islahiye districts accurately entails the geographic coordinates of the location of the damage alongside important structural data, including the year of construction and the number of storeys. This data will not only allow statistical analysis of patterns of destruction, but also the creation of scalable decision support systems to screen risks after an earthquake. The scope of this study is limited to reinforced concrete residential buildings, which constitute the dominant building typology in the affected urban areas.

In this study, officially assigned damage classifications are adopted directly, without secondary reinterpretation, to ensure consistency with national post-disaster assessment practices. Furthermore, spatial variability in ground motion demand and local site effects are explicitly considered by incorporating interpolated peak ground acceleration values and V_S30_-based site condition indicators^11,12^, while the evolution of seismic design regulations is accounted for through code assignment based on the construction period^3,13,14^. These elements collectively establish a physically grounded data foundation for the subsequent screening framework.

Despite recent advances in machine-learning-based seismic assessment, a critical limitation remains. Ozdemir and Celik^15^ suggested a Deep Residual Network for fast screening, and Yilmaz et al.^16^ used ensemble learning for multiclass damage classification. Likewise, Karampinis et al.^17^ used SHAP values for vulnerability ranking. Kumar et al.^18^ assessed the seismic vulnerability of RC educational buildings in Dhaka using SVR, Random Forest, and ANN to predict the Story Shear Ratio (SSR) as a structural risk index, with SVR achieving the highest coefficient of determination (R²=0.35). Kumar et al.^19^ developed a standalone ML framework for seismic vulnerability assessment of RC educational buildings using SVR, RFR, and ANN with Rapid Visual Assessment parameters, achieving an R² of 0.34 with SVR. While these studies establish the applicability of supervised learning to seismic vulnerability assessment, they do not incorporate spatially varying seismic demand parameters, do not employ safety-first optimization with recall-dominant objectives, and do not report subgroup performance by building height; all of which are central contributions of the present work. Nevertheless, a very important issue in these applications is the discrepancy between common ML performance measures and life-safety considerations. Most current models are designed to maximize Overall Accuracy, which tends to penalize predictions for the minority class ‘Risky’. Moreover, standalone models can neglect the intricate, nonlinear relationships between ground motion demand and structural capacity.

To overcome these issues, this paper introduces an engineering-informed, safety-first ensemble-based learning system for fast screening of building damage after an earthquake. In contrast to traditional accuracy-oriented machine learning methods, the methodology specifically focuses on life safety, minimizing false-negative predictions using cost-sensitive optimization and conservative decision thresholding. The engineering-informed design is grounded in established earthquake engineering principles; governing feature construction, selection, optimization objective, and decision thresholding; rather than embedding governing physical equations. This distinguishes the approach from physics-informed machine learning (PIML) methods, which enforce governing equations as hard or soft constraints during training. The proposed framework is validated on a massive post-disaster dataset on the Turkiye earthquakes of 2023 and has high overall performance and no false-negative predictions on high-rise reinforced concrete buildings. The proposed framework is intended as a Tier-1 post-earthquake screening tool to prioritize detailed field inspections under limited time and resource constraints.

## Study area and dataset

The ground motion records of Gaziantep show that there were very high peak ground accelerations that surpassed the design expectations in many sites. Peak ground accelerations and spectral responses were even more severe in the Islahiye district, which exhibited the highest recorded accelerations and sustained the most severe structural damage to the structure^2,9^. Surface spectral accelerations reached peak values at 1.75 g and had significant short-period de-amplification. Conversely, the surface responses of longer periods of over 0.5 s were larger than the design spectra of some soil classes by up to four times^9^. There was a high heterogeneity in the spatial distribution of building damage in Gaziantep. The destruction was most severe in the periphery: 4982 buildings were destroyed in Islahiye and 5,941 in Nurdağı^9^. The Gaziantep city center, on the contrary, had significantly fewer amounts of damage, with 44 demolished buildings only. This spatial distribution shows a very visible correlation with both epicentral distance and local soil conditions, such that a dataset of wide seismic demand levels is obtained.

**Fig. 1:**
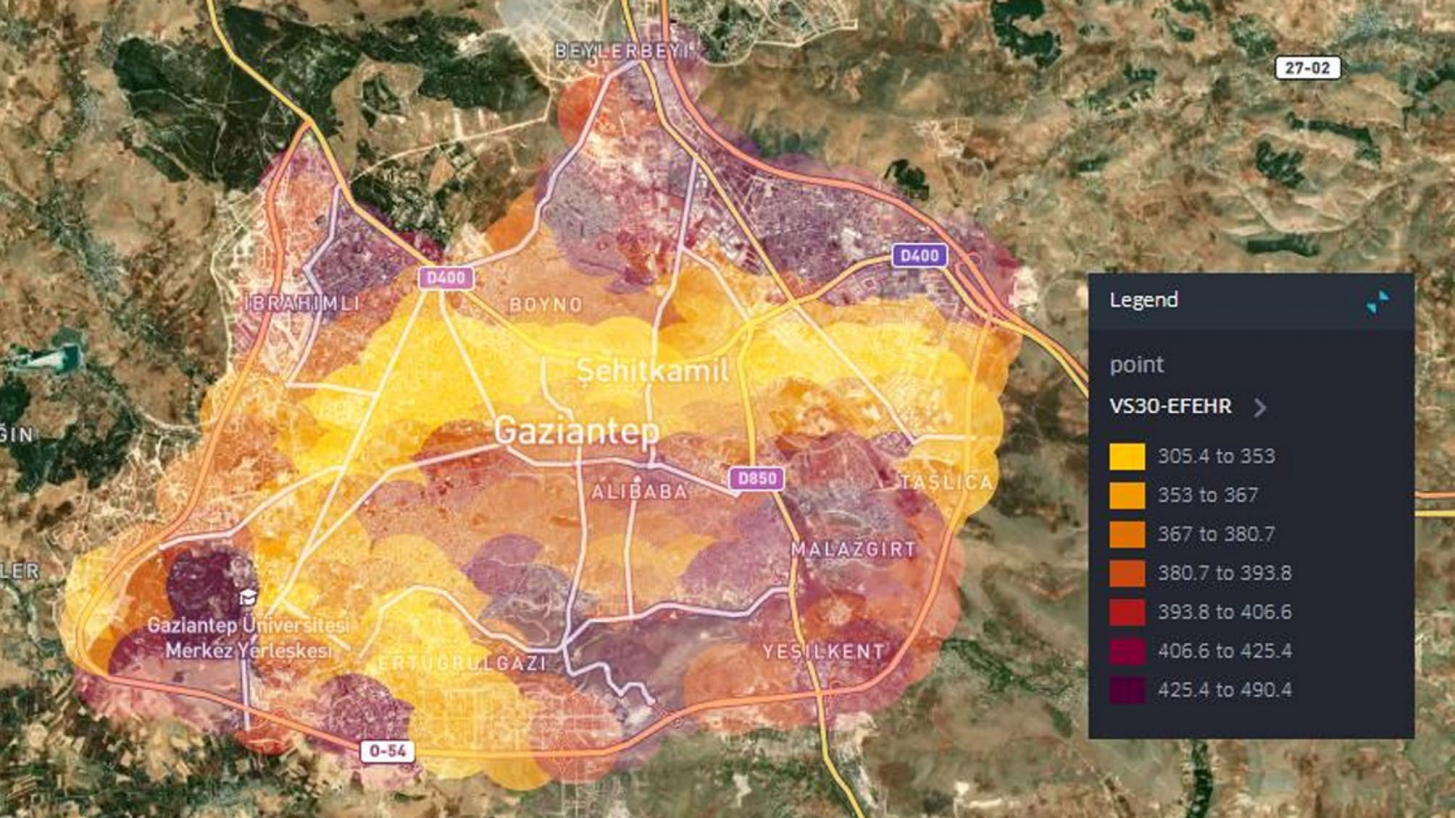
V_S30_ map of Gaziantep showing V_S30_ distribution^20^. (GAUS 2025, Sustainable and Smart Mobility Strategy Plan for Gaziantep).

Geotechnical investigations further revealed considerable spatial variability in V_S30_ values, representing the average shear-wave velocity in the upper 30 m of the soil profile. Site classifications predominantly corresponded to NEHRP Classes B (very dense soil or soft rock) and C (moderately dense to dense sands, gravels, or hard clays). Nevertheless, site-specific seismic response analyses identified significant amplification in areas underlain by thick alluvial deposits, with soft-soil amplification factors exceeding 1.6^10^ The geographical layout and the V_S30_ distribution of the study area are illustrated in Fig. 1.

**Fig. 2:**
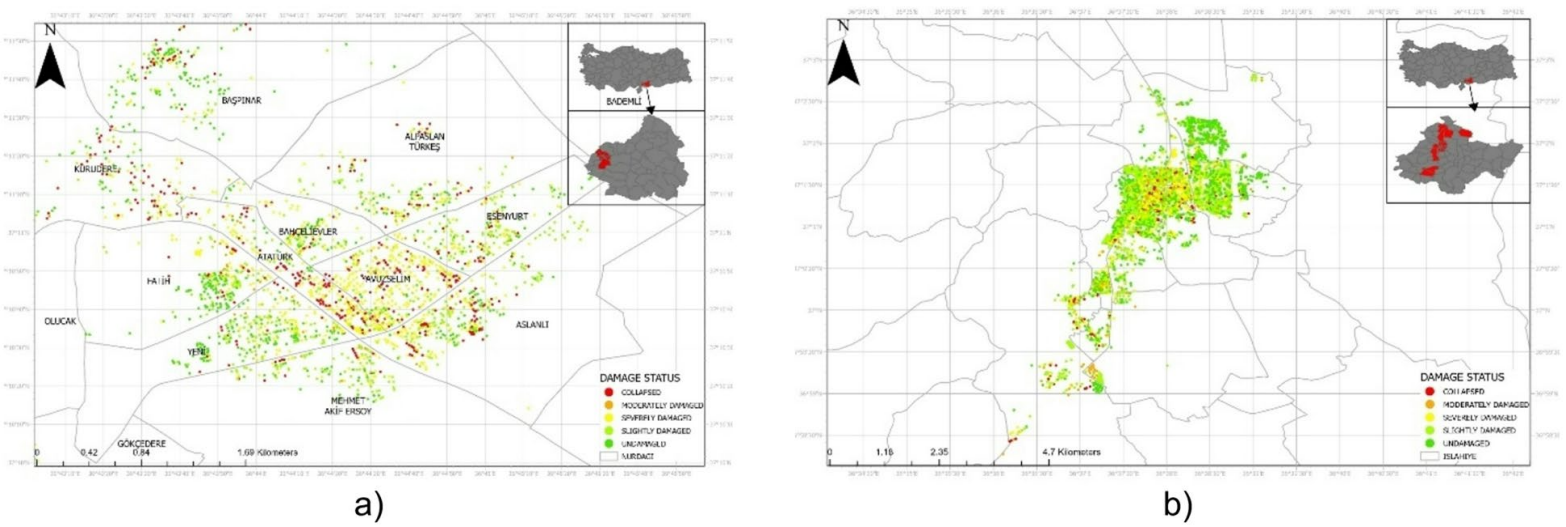
Point-based spatial distribution of damage states in (**a**) Nurdağı and (**b**) Islahiye.

**Fig. 3:**
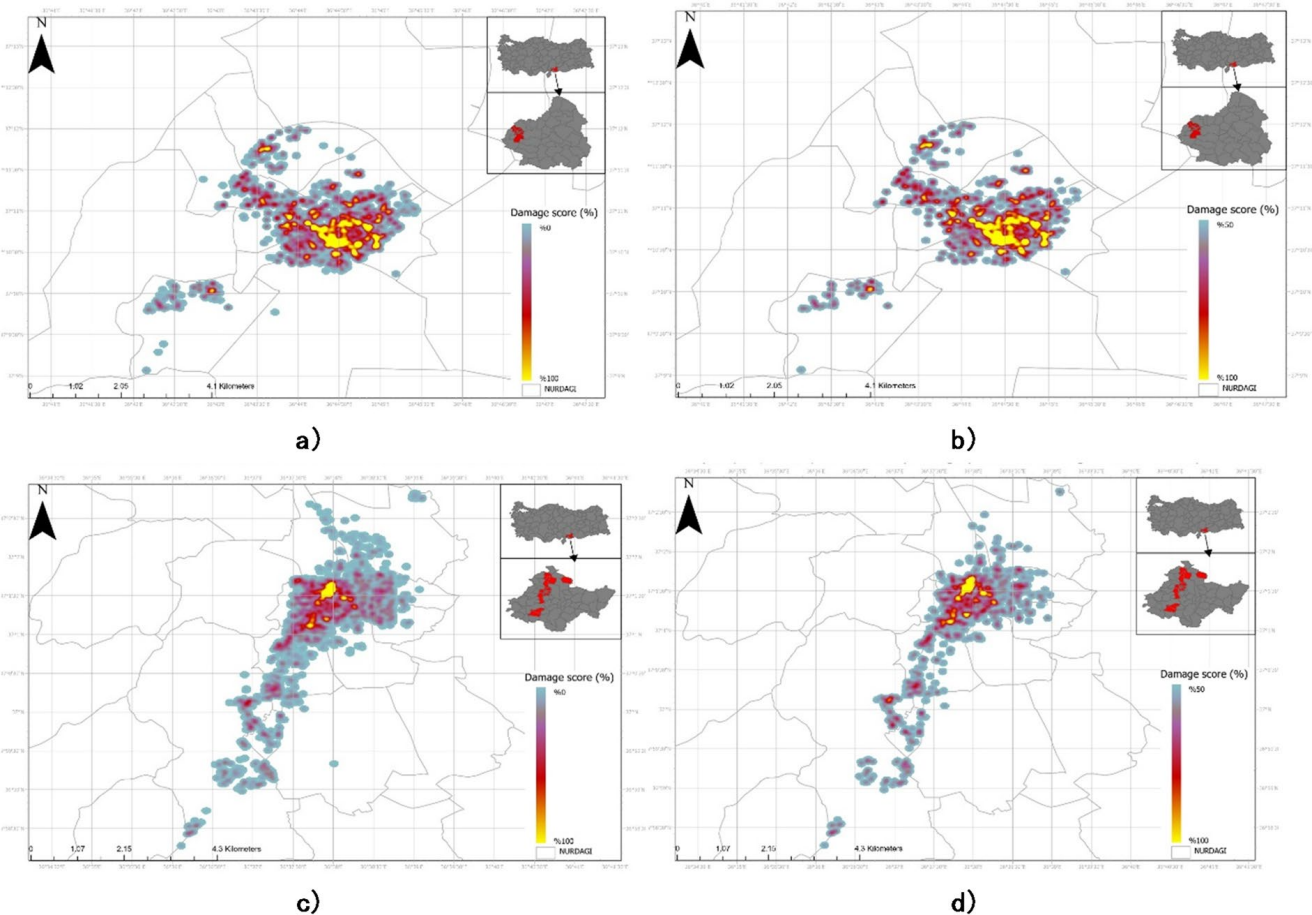
Spatial heat maps showing damage concentrations. (**a**, **b**) Collapsed buildings; (**c**, **d**) Unusable (collapsed + severe/moderate) buildings.

**Fig. 4:**
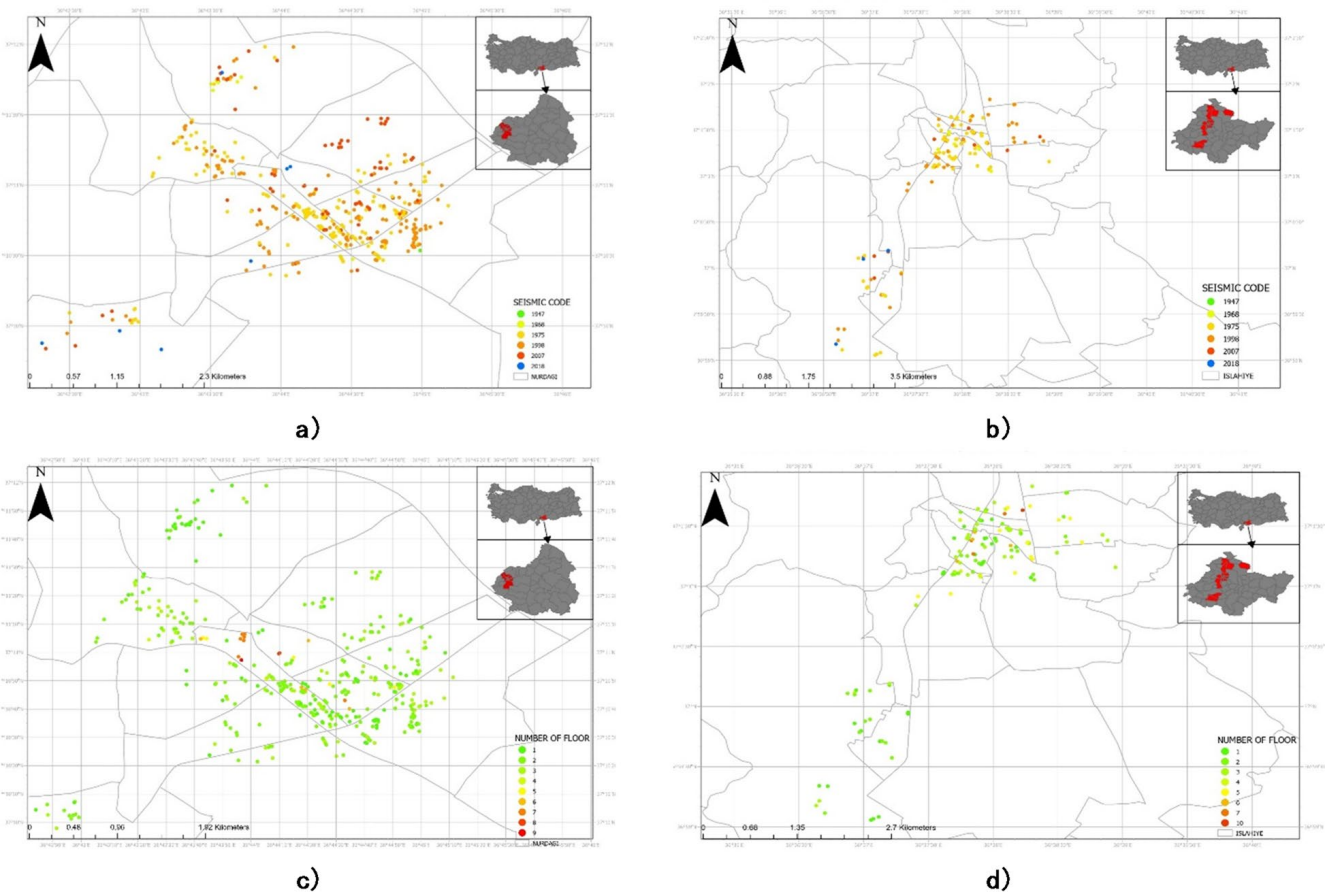
Distribution of collapsed buildings categorized by (**a**, **b**) Seismic Design Code and (**c**, **d**) Number of Storeys.

**Fig. 5:**
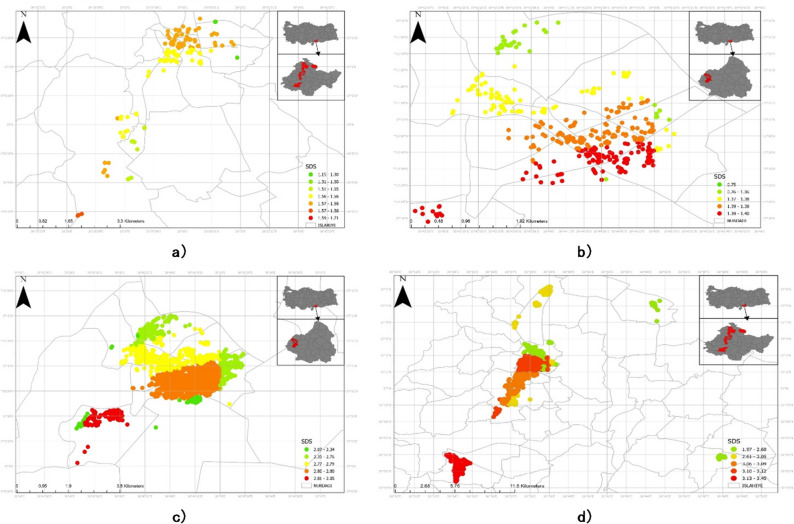
Distribution of collapsed buildings overlaid on Design Spectral Acceleration (SDS) maps for DD-2 (**a**, **b**) and DD-1 (**c**, **d**) hazard levels.

Figure 2 presents the point-based spatial distribution of damage states for reinforced concrete buildings in the central neighborhoods of Nurdağı and Islahiye, respectively, revealing pronounced clustering of collapsed and severely damaged buildings within the urban cores. The areas with the concentration of the collapsed buildings are also highlighted by the heat maps in Fig. 3, where the hotspots of the damage become visible on the most densely developed territories of the two districts. The location of high-damage areas when the non-functional buildings (collapsed and grossly damaged as well as moderately damaged (Fig. 3) are taken into account, the area of high-damage buildings increases dramatically, and it implies that the loss of functionality covers a much larger area of the urban fabric than that represented by buildings that have been completely collapsed. Figure 4 shows how collapsed buildings are distributed based on the seismic code that was used at the time of construction so that most collapses are on buildings that were designed using older seismic codes, whereas those built using more recent seismic codes record very few collapses. The building height effect is investigated in Fig. 4, which reveals that the collapsed structures in both Nurdagi and Islahiye are mostly low- and mid-rise in nature and show that there is a critical range of vulnerability that is dependent on the typical construction methodologies rather than extreme building heights. Lastly, the correlation between seismic demand and the presence of a collapse can be shown in Fig. 5, whereby collapsed buildings in both districts are always concentrated at the higher design spectral acceleration (SDS) under DD2 and DD1 hazard levels, which indicates the joint impact of high seismic demand and structural vulnerability on damage pattern.

Field checks revealed that significant deficiencies in material quality were identified, which contributed largely to structural failures that have been observed. The recorded concrete compressive strengths in most broken or grossly damaged buildings were found to be 3.8 to 13.8 MPa, which is significantly below the minimum of 20 MPa, as provided by the present-day design codes^1^. Moreover, brittle fracture characteristics and a lack of ductility were found in nearly three-quarters of the surveyed reinforcing steel, and often did not meet the requirements of TS708 because of inadequate chemical composition ^1^.

**Fig. 6:**
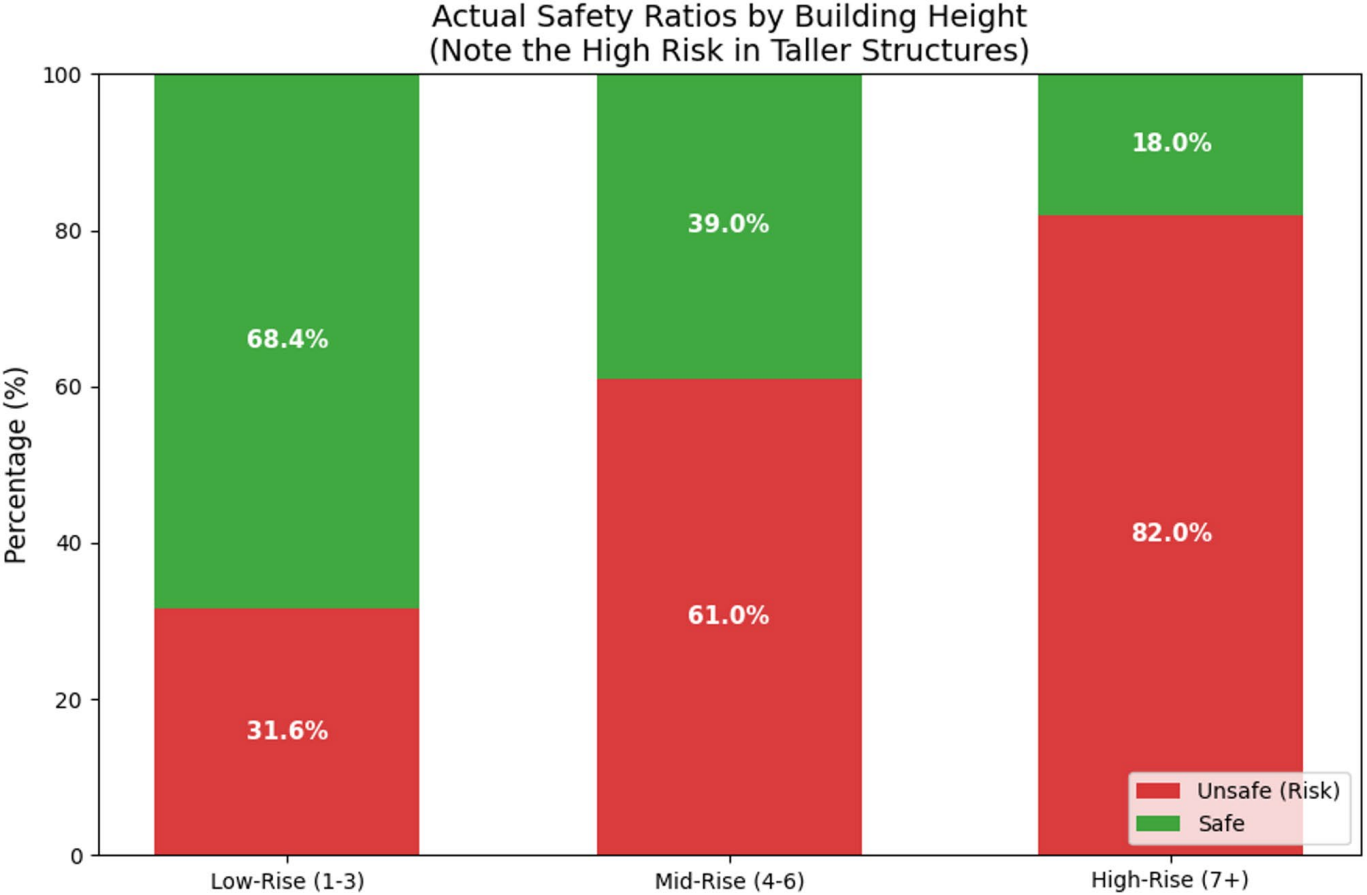
Observed damage distribution by building height, highlighting the disproportionate contribution of mid- and high-rise structures to severe damage.

Lack of transverse reinforcement detailing was one of the most common structural weaknesses in the damaged building stock in Gaziantep. Poor distances between stirrups and common use of 90-degree hooks instead of 135-degree hooks required by Turkish Seismic Design Codes between 1975 and 2018 supported longitudinal reinforcement buckling, and later crushing of the concrete core^1^. Figure 6 gives a general idea of the frequency of observed destruction by the height of the building, with no causal relationships.

### Assessment methodology

The labels of target variables used in this study (Safe versus Unsafe) were obtained with the help of the Ministry of Environment, Urbanization, and Climate Change (MoEUCC) algorithm, the Crisis Damage Assessment (CDA). As is reported by Atasever et al.^21^, this protocol is not limited to a mere visual examination, but is a hierarchical approach, which is based on mechanical grounds, and attempts to discriminate between the occurrence of vertical load-bearing failure and those structures that are still sufficiently stiff to allow life safety.

The CDA process has a two-phase evaluation course. The initial phase is an outside scrutiny that is concerned with global stability. Immediately, buildings with total structural failure, partial collapse of the storeys, and residual inter-storey drift of 3.0% or more, or rigid-body rotation of more than 4° all fall under the Collapsed or To Be Demolished category^21^.

When a structure is considered globally stable, then there is a second-stage internal inspection done at the component level. At this stage, the assessment of vertical load-bearing members (columns and shear walls) rather than the beams is given preference, specifically, the well-known strong beam-weak column failure mechanism.

The states of damage are measured in relation to physically imposed material limits. Type B (Yielding) damage is defined by a crack width of 0.5 < w < 3.0 mm in the concrete cover or slabs that are spalling away in reinforcement, and has the core confinement intact. Instability (Type C) is associated with minimal reinforcement buckling, which is the threshold of successful confinement. Type D (Loss of Capacity) refers to extreme damage, such as core crushing, reinforcement rupture, or high buckling, and is linked with total axial load-carrying failure.

The algorithm uses these inputs to classify global damage. A structure is defined as being “Heavily Damaged” (Risky) when it has a residual drift of at least 1.0%, has one Type D structural member, or has two Type C structural members. Buildings with a damage level under these defined levels are found in the category of the Structures that are considered as “Moderately” and “Slightly Damaged” (Safe/Repairable). This classification scheme has a low revision rate of 0.75% in a small sample of 2.3 million reported by Atasever et al.^21^, which supports its reliability. However, while formal measures of inter-rater agreement, such as Cohen’s Kappa, are not provided for the CDA protocol, the remarkably low rate of revision among assessments carried out by independent engineering teams in different cities constitutes compelling proof of high inter-rater agreement. International earthquake Rapid Visual Assessment programs frequently report disagreement rates of about 5–20% (for example, ATC-20 quick tagging protocols in the U.S.); in contrast, the rate of 0.75% revisions in the case of the mechanics-based quantitative CDA protocol is significantly lower. The binary classification used in the CDA protocol categorizes Undamaged and Slightly Damaged structures as Safe and Moderately Damaged, Severely Damaged, and Collapsed buildings as Unsafe. The inclusion of Moderately Damaged buildings in the Unsafe category is justified on three counts: (i) engineering considerations – Moderately Damaged buildings have suffered damage that affects their structural safety sufficiently that they cannot be occupied until repairs are performed and confirmed, and could undergo progressive failure when subjected to shock loadings from after-shocks; (ii) regulatory considerations – the MoEUCC protocol identifies Moderately Damaged buildings as needing to be evacuated; and (iii) conservative triage philosophy – as a Tier-1 instrument, an inclination to flag buildings for further inspection is to be preferred over prematurely clearing them.

The paper is based on a post-earthquake damage assessment database assembled after the 2023 Kahramanmaraş earthquake sequence. The Ministry of Environment, Urbanization, and Climate Change provided the damage assessment data of the Nurdagi district and the Islahiye district on December 9, 2024. The dataset comprises geographic position, condition of damage status, building year of construction, structure system, number of storeys, and neighbourhoods of all the reviewed buildings in the two districts.

The first database contained 13,292 buildings. To analyze data, 7292 reinforced concrete buildings, having all the information about the time of construction, the number of storeys, geographic position, and the condition of damage, were drawn into the analysis. Status of damage, classification of structural systems, and geometrical attributes were taken as they are presented by the Ministry, and no secondary evaluation or reconsideration of the damage states was carried out.

The physical parameters are summarized in Table 1 in terms of the statistical distribution. The data is indicative of a high seismic demand environment, whereby spectral design acceleration (SDS) values are 1.44 g with high values of peak ground acceleration (PGA) of up to 0.60 g in near-fault regions. The property is mostly low- to mid-rise, with a mean storeys height of 2.3, which aligns with the pre-eminence of residential buildings in peripheral areas. The construction year covers one century (1923–2023), and the average construction year is 1999, which is related to one of the most significant shifts in the Turkish seismic design regulations.

**Fig. 7:**
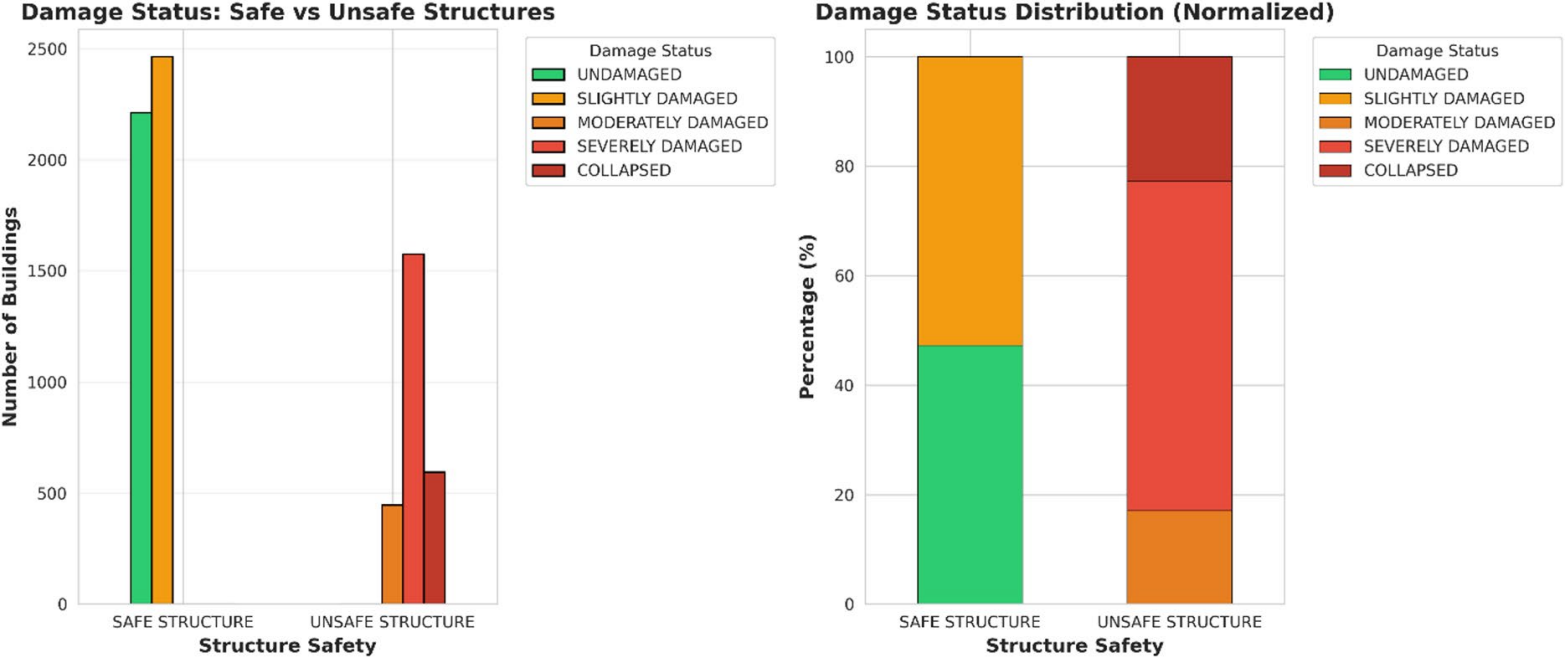
Safe vs. unsafe structures.

The sample exhibits a moderate class imbalance. As shown in Fig. 7, most of the structures (64.10%) were categorized under the safe category (Green) and the remaining 35.90% under the unsafe category (Red). This approximately 1.8:1 Safe-to-Unsafe ratio requires the Cost-Sensitive Learning strategy described in Sect.  3.4 to ensure that the model does not skew predictions toward the majority class.

**Table 1:** Descriptive statistics of the dataset.

Feature	Description	Unit	Mean	Min	Max	Std. Dev.
PGA	Peak ground acceleration	g	0.52	0.24	0.60	0.04
SDS	Short-period spectral acc.	g	1.44	0.72	1.70	0.12
V_S30_	Shear wave velocity	m/s	455	229	847	90
STOREYS	Building height	Count	2.3	1	11	1.6
YEAR	Year of construction	Year	1999	1923	2023	13

The national earthquake hazard data published by the Disaster and Emergency Management Authority were used to obtain site-specific seismic demand parameters^22^. The AFAD earthquake hazard map had a value of Peak Ground Acceleration (PGA) at DD-2 (475-year return period) and DD-1 (2475-year return period) earthquake ground motion levels. In this framework, spatially varying seismic demand is represented using the interpolated design spectral acceleration (S_DS_) and Peak Ground Acceleration (PGA) corresponding to the DD-2 (475-year return period) hazard level from the official AFAD national seismic hazard map, rather than event-specific ShakeMap intensities. This operational choice was made for two primary reasons. First, from a structural engineering perspective, the inherent vulnerability of the building stock is fundamentally coupled with the design forces anticipated at the location; referencing the code-basis DD-2 hazard level establishes a consistent, measurable baseline for evaluating expected structural capacity. Second, from an operational triage perspective, a predictive framework reliant on pre-computed, static national hazard maps can be deployed instantaneously following an earthquake. In contrast, high-fidelity event-specific ShakeMaps require the assimilation of strong-motion station recordings and macroseismic reports, which can take days to stabilize in regions with sparse instrument coverage. Therefore, utilizing the DD-2 proxy strictly aligns with developing a “zero-hour” rapid decision-support tool.

Given that hazard values are available at the discrete grid points, PGA values of a particular building location were interpolated with the help of the Inverse Distance Weighting (IDW) interpolation process. Six nearest hazard grid points were taken into account with regard to each building coordinate. Kriging was not used because the IDW method was chosen to be more transparent and faster to verify the interpolation process.

The values of V_S30_ were used to represent local site conditions by using those acquired in the European Facilities for Earthquake Hazard and Risk^20^ database. The V_S30_ values were extracted, and classes of soils were applied to each building site. Based on the interpolated PGA values and the assigned soil classes, short-period design spectral acceleration coefficients (SDS) of DD-1 and DD-2 levels of the earthquake were determined.

**Fig. 8:**
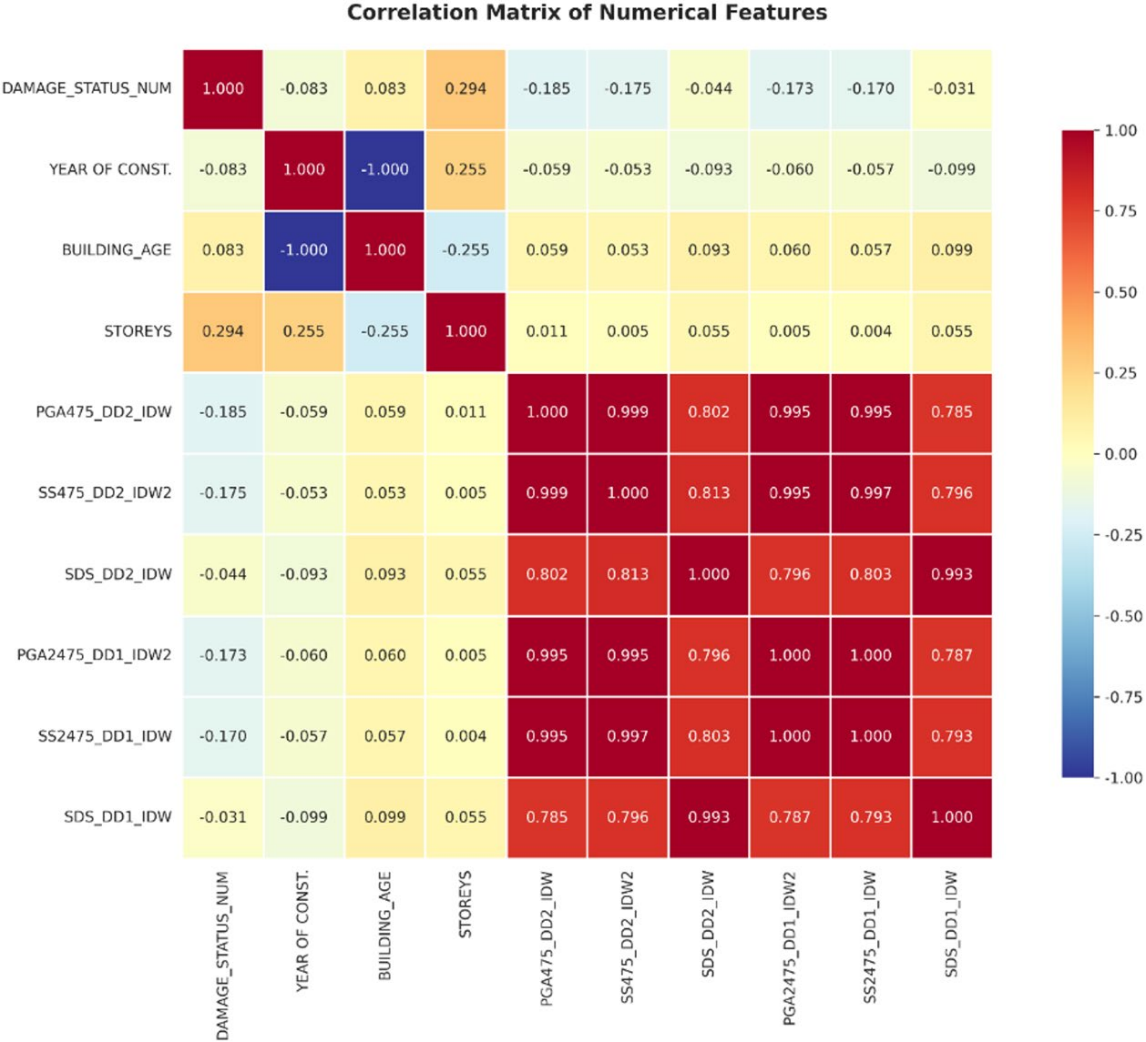
Correlation heatmap (raw data).

The Pearson correlation table (Fig. 8) offers important information that was used to guide the further feature engineering policy. This analysis can be summarized into three important observations.

To begin with, there is high multicollinearity among the measures of seismic intensity. In the bottom-right corner of the matrix, there are almost perfect correlations (*r* > 0.99) between multiple spectral acceleration parameters (e.g., PGA, Ss, and SDS). Such a high redundancy implies that the addition of multiple seismic indices would add unwarranted noise and increase the issue of multicollinearity. Based on this, SDS_DD2_IDW was retained as the primary spectral demand feature due to its direct relevance to code-based design provisions for RC frames, while PGA_DD2_IDW was retained as a complementary broadband intensity measure capturing near-fault impulsive effects. All other correlated spectral parameters (PGA_DD1, Ss_DD1, Ss_DD2, SDS_DD1) were removed to eliminate redundancy.

Second, building height emerges as one of the strongest correlates of damage. Of the structural characteristics, the number of storeys (STOREYS) shows the most positive relationship with the status of damage (*r* = 0.294). The observation correlates with the observations of post-event fields, which emphasized the increased vulnerability of the frame made of reinforced concrete (mid to high-rise) to resonance in the course of the examined earthquake.

Lastly, the absolute values of the correlation coefficients of single features to damage status are indicative of the highly non-linear character of the correlation between seismic demand and structural capacity. This complexity can not be appropriately represented using linear modeling techniques, which is why the use of non-linear Gradient Boosting frameworks, including the LightGBM architecture, is warranted in this paper.

The final retained feature set consists of seven variables: STOREYS, YEAR, PGA_DD2_IDW, SDS_DD2_IDW, V_S30_, STOREYS×YEAR, and YEAR×PGA. These names are used consistently across all subsequent sections, tables, figures, and SHAP analyses. PGA_DD2_IDW and SDS_DD2_IDW are complementary rather than redundant: PGA captures broadband near-fault impulsive effects, while SDS captures the short-period spectral demand governing RC frame response.

Tables 2 and 3 give a summary of the settlement of post-earthquake damage conditions in accordance with seismic design codes, periods, and height of the building, respectively. These findings indicate that later code stages and more storeys are linked to more severe and collapse-level damage, which highlights the significance of control development as well as the structure layout in measured seismic performance.

**Table 2:** Distribution of building damage states by seismic design code period.

Damage state	< 1947	1947	1953	1961	1968	1975	1998	2007	2018	Total
Collapsed	–	1	2	1	16	262	213	87	12	594
Moderately Dmg.	–	–	4	5	24	168	113	87	45	446
Severely Dmg.	–	4	9	8	40	694	430	314	76	1575
Slightly Dmg.	–	2	12	25	84	1013	674	539	116	2465
Undamaged	7	1	–	4	67	735	737	501	160	2212
Total	7	8	27	43	231	2872	2167	1528	409	7292

**Table 3:** Distribution of building damage states by number of storeys.

Damage State	1	2	3	4	5	6	7	8	9	10	11	Total
Collapsed	125	276	135	12	21	10	11	2	1	1	–	594
Moderately Dmg.	91	167	72	16	18	29	38	8	5	1	1	446
Severely Dmg.	209	671	295	90	54	74	84	76	21	–	1	1575
Slightly Dmg.	759	1173	328	79	40	31	27	11	13	4	–	2465
Undamaged	1057	899	199	21	10	26	–	–	–	–	–	2212
Total	2241	3186	1029	218	143	170	160	97	40	6	2	7292

## Methodology

**Fig. 9:**
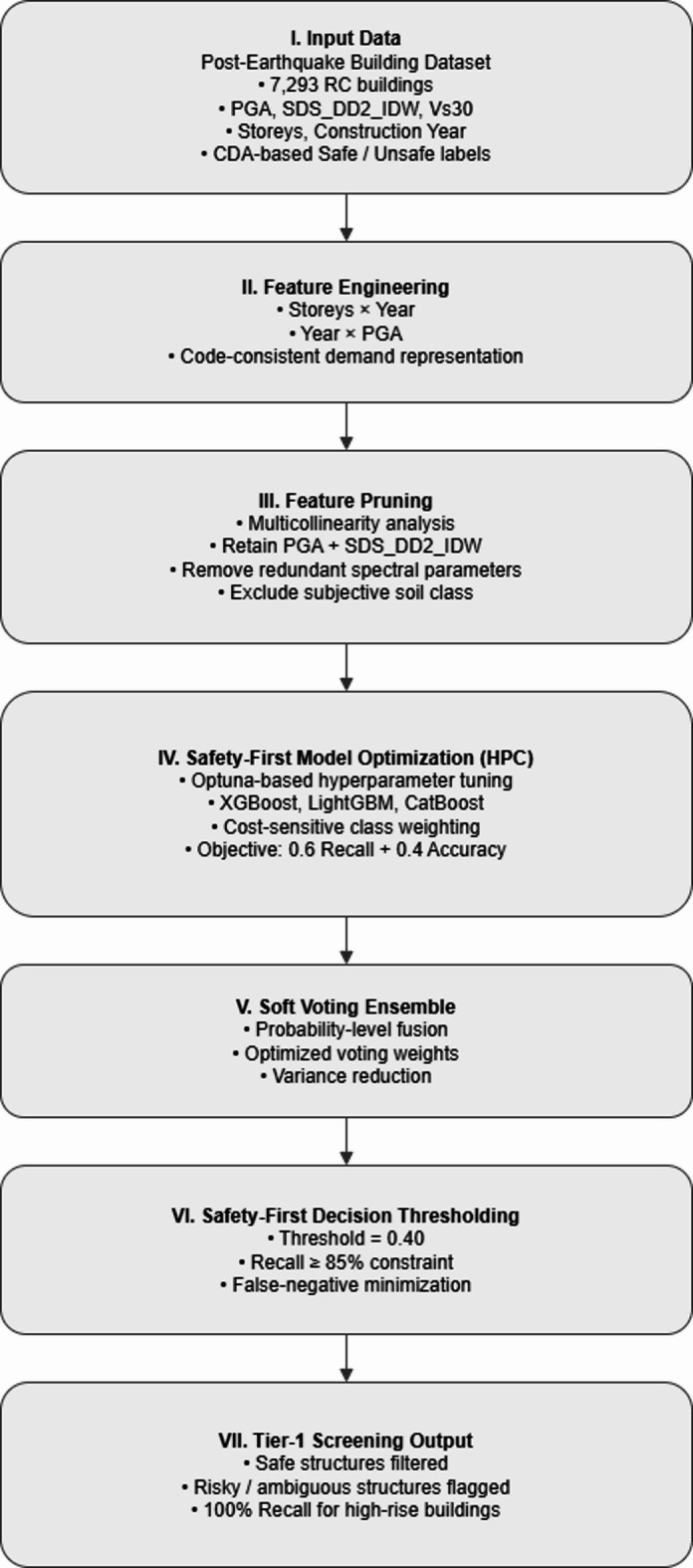
The proposed methodology flowchart.

The study has a multi-stage workflow that is focused on life safety, comprising four fundamental methodological phases and the supporting optimization and decision stages, as illustrated in Fig. 9. Random sampling was employed to divide the dataset in 80% training and 20% testing in order to maintain the class proportion (Safe/Unsafe ratio). Engineering-informed, as used in this study, denotes a design philosophy in which every stage of the data-driven pipeline — feature construction, feature selection, optimization objective, and decision thresholding — is constrained by and aligned with established principles of earthquake engineering and life-safety codes, rather than being determined by purely statistical criteria. This approach does not embed governing equations into the learning architecture, which distinguishes it from physics-informed machine learning (PIML) in the strict sense.

### Data splitting and stratification

The dataset was partitioned into 80% training and 20% testing subsets using stratified random sampling to preserve the Safe/Unsafe class proportion. While this random split is retained as the primary validation protocol for comparability with existing literature (where random partitioning remains the dominant strategy in post-earthquake damage classification studies), a supplementary spatial robustness analysis is reported in Sect.  4.4. That analysis employs geographically independent validation by treating the two districts (Nurdağı and Islahiye) as separate training and test domains, ensuring that no building in the test fold shares a neighbourhood with any building in the training fold. Though conducting full spatiotemporal cross-validation is not feasible since the data is limited to two regions only, the leave-one-region-out cross-validation used in the current research represents the most stringent level of geographic stratification that can be achieved, ensuring spatial independence between the training and validation sets.

### Feature engineering and pruning

The traditional data-driven models usually consider the input features as independent variables; nevertheless, the seismic risk is essentially controlled by the correlation between structural capacity and seismic demand. Engineering-informed interaction features were hand-coded to capture these latent dependencies.

Interaction term 1 (Storeys × Year) is the cumulative weakness of taller buildings built using outdated seismic design codes. An example is a 10-storey building constructed in 1980, which has a much greater risk compared to a 2-storey building of the same time, which is usually hard to discern in purely linear formulations. A second interaction term (Year × PGA) is indicative of the demand capacity relationship with the difference between aging structures, which are under intense ground movements (high risk), and structures that are in areas of less seismic demand (low risk).

The initial exploratory research showed that the categorical type of variable, the Soil Class, added a significant amount of noise to the study, and to a great extent because of subjective field-based classification. A sensitivity study (an ablation experiment) revealed that the omission of this categorical parameter and using instrumental parameters (PGA and V_S30_) to replace it increased the Area Under the Curve (AUC) of the model from 0.80 to 0.83. Based on this, the last model takes a small and direct feature set that is based solely on physical measured parameters.

### Ensemble learning architecture

To address the bias and variance associated with single-algorithm models, a Soft Voting Classifier ensemble was developed. This architecture integrates the complementary strengths of three state-of-the-art Gradient Boosting Decision Tree (GBDT) algorithms:

1. **XGBoost**: Selected for its strong regularization capabilities (L1/L2), which effectively mitigate overfitting during training^23^.
2. **LightGBM**: Employed for its leaf-wise tree growth strategy, offering high computational efficiency and scalability on large datasets^24^.
3. **CatBoost**: Incorporated due to its symmetric tree structure and its ability to capture complex non-linear interactions with minimal preprocessing requirements^25^.

The final prediction probability (P_final_​) is computed as a weighted average of the individual predictions produced by these base learners.

$${{\mathrm{P}}_{{\mathrm{final}}}}\,=\,\sum {{{\mathrm{w}}_{\mathrm{i}}}} \cdot{\mathrm{Pi}}\left( {\mathrm{x}} \right)$$

where w_i_ represents the optimized weight for each algorithm.

### Safety-first optimization via HPC

The TRUBA HPC cluster was tested on Optuna, with the hyperparameters being optimized. In contrast to the traditional grid search algorithms, which mainly maximize Accuracy, a tailored “safety-first” purpose was formulated as

$${\mathrm{Score}}\,=\,0.{\mathrm{6}} \times {\mathrm{Recall}}\,+\,0.{\mathrm{4}} \times {\mathrm{Accuracy}}$$

It can be seen that the weighting scheme chosen in the custom objective function (0.6 x Recall + 0.4 x Accuracy) represents the inherently asymmetric cost of misclassification in seismic risk screening. In earthquake engineering and disaster mitigation, a false negative (not identifying that an unsafe structure is safe) has a potentially disastrous effect on the loss of life, whereas a false positive (making an extra inspection and an increase in logistical load) has a minor impact. In line with this, Recall was specifically overweighted over Accuracy so as to appropriately fit the optimization procedure with life-safety goals. The initial sensitivity analysis results suggested that the model was not sensitive to Recall-dominant weightings in the 0.55–0.70 range, which supports the 0.6 / 0.4 balance being found as a robust yet conservative calibration and not an arbitrary choice.

This formulation is an explicit punishment of false negatives (i.e., missed hazardous buildings) that are harsher than false positives. Moreover, there was a hard constraint, according to which the results of any trial that gave Recall less than 85% were penalized and did not enter into consideration. After the training of the models, the decision threshold was tuned empirically. The default value of 0.50 was changed to 0.40, therefore, creating a more risk-averse decision line that would rather raise the ambiguous structures to be inspected instead of being considered as safe. All seven features; including the two engineered interaction terms (STOREYS×YEAR and YEAR×PGA); were standardised using z-score normalisation (StandardScaler) fitted exclusively on the training set and applied to the test set without refitting. Although the three base learners are gradient boosting decision tree (GBDT) algorithms whose split-point selection is invariant to monotonic feature transformations, standardisation was applied uniformly across all features including the interaction terms to ensure consistent scaling within the soft-voting probability aggregation step. No separate normalisation of the interaction terms was performed beyond this unified scaler. The Bayesian hyperparameter search was conducted using Optuna with the Tree-structured Parzen Estimator (TPE) sampler on the TRUBA HPC cluster over a 6-hour optimisation run. Each candidate configuration was evaluated using stratified 5-fold cross-validation on the training set (*n* = 5,833), with the custom safety-first objective (0.6×Recall + 0.4×Accuracy) computed as the mean across folds; any trial yielding a mean Recall below 85% was penalised and excluded. The 15 hyperparameters, spanning the internal parameters of all three base learners (n_estimators, max_depth/leaves, learning_rate, subsample, colsample_bytree) and the ensemble-level voting weights (w_XGB, w_LGBM, w_CAT) and class weight (unsafe_w), were optimised jointly in a single search space. The best performing trial scored 0.787 in objective, meeting the expected optimal parameter setting shown in Table 4. Calibration through Platt scaling or isotonic regression was not used in this work. The decision threshold value set is fixed at 0.40 based on Table 5, and does not rely on well-calibrated probabilities. Furthermore, it should be mentioned that the ordered boosting in CatBoost results in similarly well-calibrated predictions, as compared with other GBDT models. This is evidenced by the Brier score of 0.186 (see Table 6), which is considerably smaller than the base score of 0.230, and hence demonstrates the quality of probability predictions.

The results of optimization are summarized in Table 4 yield three important insights concerning the internal behavior of the model. First, the Optuna search converged to a conservative model architecture (i.e., very low learning rates, e = 0.0068) and shallow tree depths (maximum depth < 4). This implies that noisy seismic data is robustly generalized using a slow and highly regularized scheme that reduces overfitting to outliers. Second, the ensemble voting weights define XGBoost as the most significant contributor (w_XGB_ = 1.94), which is probably related to the fact that it manages the issue of class imbalance effectively through the optimized scalepos_weight parameter (3.45). Lastly, the high class weights of the Unsafe category mathematically enforce the safety-first paradigm, which obliges the optimizer to severely punish the failure to classify hazardous buildings, but not the false alarms.

**Table 4:** Optimized hyperparameters.

Hyperparameter	XGBoost	LightGBM	CatBoost
Number of estimators	584	258	444
Learning rate	0.0068	0.0199	0.0104
Max depth/leaves	4 (Depth)	31 (Leaves)	4 (Depth)
Class weight (unsafe)	3.45	3.45	3.45
Voting weight (w)	1.94	1.19	1.72

**Table 5:** Threshold optimization analysis.

Threshold	Accuracy	Recall (sensitivity)	Decision logic
0.30	0.581	0.937	Extremely conservative
0.35	0.618	0.908	Very conservative
0.40	0.657	0.889	Selected optimal (safety-first)
0.45	0.688	0.843	Balanced
0.50	0.716	0.811	Standard (high risk)
0.60	0.758	0.727	Unsafe for screening

**Table 6:** Final performance metrics.

Metric	Value	95% CI	Interpretation
Unsafe recall (sensitivity)	88.91%	[85.9%, 91.5%]	Successfully identifies 9 out of 10 risky buildings.
Safe recall (specificity)	52.78%	[49.5%, 56.0%]	Reflects intentional safety-first trade-off
Precision (unsafe)	51.27%	[48.0%, 54.6%]	~ 1 in 2 flagged buildings is truly unsafe
Precision (safe)	89.49%	–	Buildings cleared as safe are highly reliable
F1-Score (unsafe)	0.6503	–	Reflects precision–recall trade-off
AUC score	0.8122	[0.788, 0.837]	Strong discrimination capability
Accuracy	65.73%	[63.2%, 68.2%]	Moderate, due to the intentional safety bias.
Brier score	0.1863	–	Well below the naive baseline of 0.230
Missed detections	58 / 523	–	Only 11% of risky structures were missed.

### Handling imbalanced data and weighted training

Table 5 summarizes the trade-off between Accuracy and Recall (Sensitivity) at the various decision thresholds. One important finding is that the default threshold of 0.50 that is widely used in classification tasks leads to a Recall of 81.1 only. Considering seismic screening, it would mean that almost 19% of the dangerous structures are classified as safe, which is an unacceptable threat to the safety of life.

On the flip side, a threshold of 0.30 yields the highest Recall of 93.7% at the cost of Accuracy of 58.1, which would place an undue strain on municipal resources since the false positives would be very high. Thus, the best compromise was 0.40 chosen as a threshold. This operating point provides the model with a Recall of about 89%, thus providing a reasonable “safety margin” to capture borderline cases without an excessive false positive rate to engineers who will be tasked with conducting the inspection.

## Results

### General performance

**Fig. 10:**
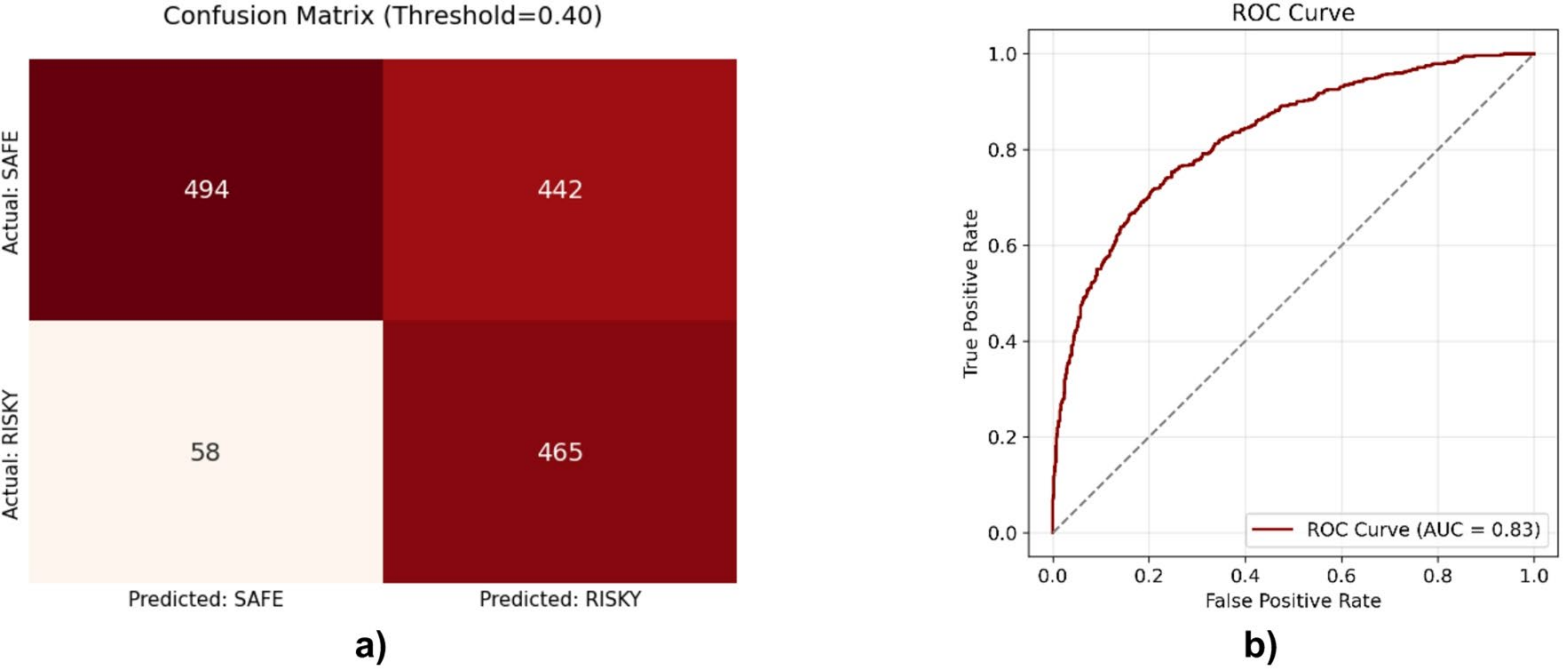
(**a**) Confusion matrix. (**b**) ROC curve.

The results evaluated on the unseen test set confirm the framework’s generalization capability, as illustrated in Fig. 10. An AUC value of 0.8122 demonstrates a strong capacity to discriminate between safe and unsafe structural signatures, independent of the selected decision threshold. More importantly, the disparity between the moderate Accuracy (65.73%) and the high Unsafe Recall (88.91%) quantitatively reflects the intended “safety-first” calibration. By explicitly penalizing false negatives during training, the model reshapes the error distribution, tolerating a higher rate of false positives to ensure that nearly nine out of ten hazardous buildings are correctly identified. In practical terms, out of 523 unsafe structures in the test set, only 58 were misclassified, thereby substantially reducing the subset of buildings requiring urgent manual inspection. 95% confidence intervals are shown in Table 6 for all primary measures. The lower limit for the Confidence Interval of Recall is 85.9%. This shows that despite having sampling variation, the model maintains consistent superiority to the 85% hard requirement used in optimization. Also, the confidence interval for the AUC measure of [0.788, 0.837] is evidence of consistent discriminative ability.

Recall, Specificity, Precision, and Accuracy confidence intervals computed using the Clopper-Pearson exact method. AUC confidence interval computed using the DeLong method (Hanley & McNeil, 1982). Precision (Safe) = TN/(TN + FN) = 494/552. Naive Brier score baseline = 0.230 (predicting class prevalence for all buildings).

**Fig. 11:**
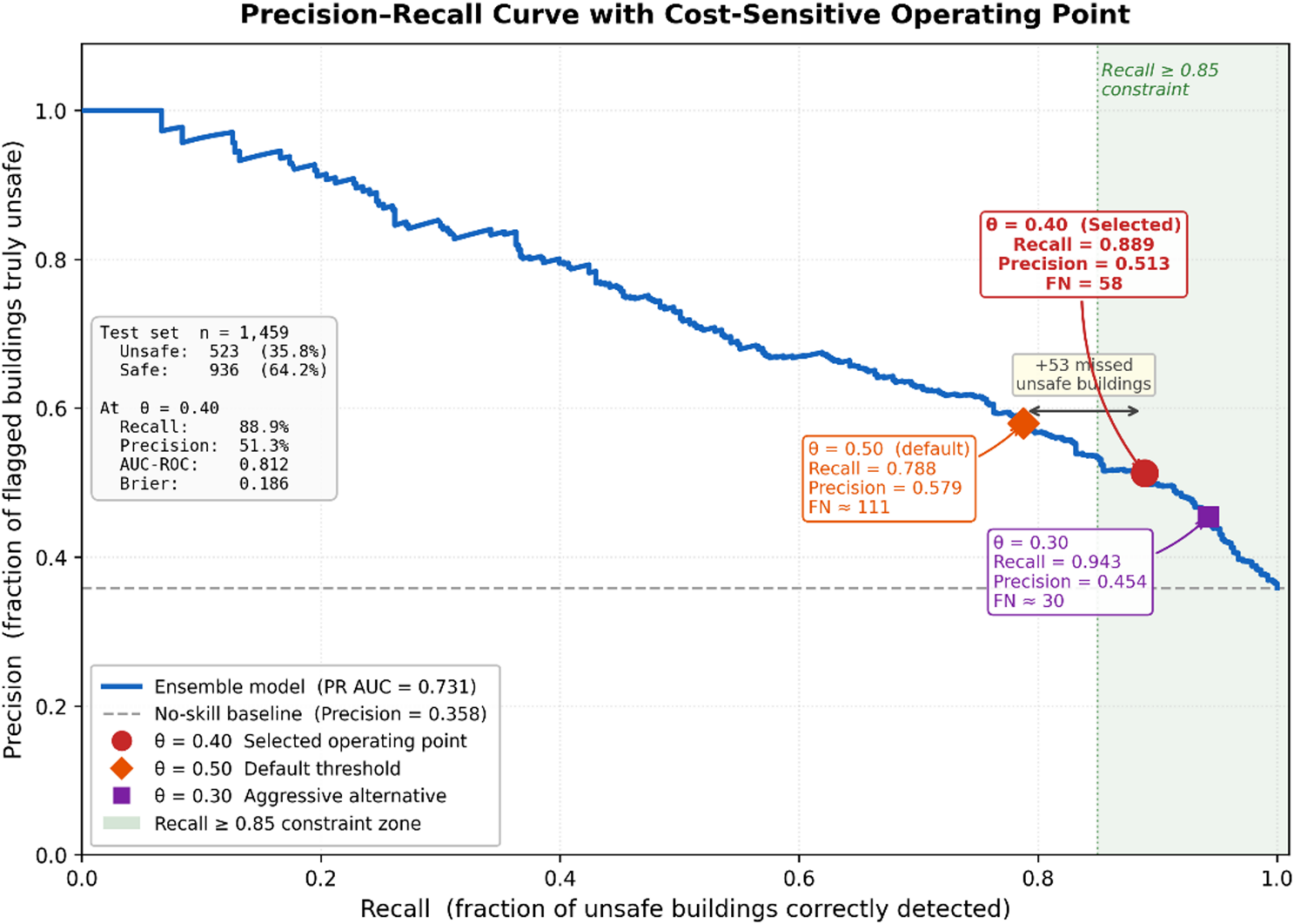
Precision–recall curve for the safety-first ensemble on the independent test set.

Figure 11 shows that by setting θ = 0.40, 53 more unsafe buildings can be detected in comparison to when the threshold value is set at its default value θ = 0.50, and at the same time, the constraint Recall ≥ 85% will still be satisfied.

### Performance on critical high-rise structures

The result of the subgroup analysis shown in Table 7 represents the most significant contribution of this study to disaster risk mitigation. The model achieved a perfect Recall of 100% for high-rise buildings (6 + storeys) and 97.1% for mid-rise structures. A conservative decision threshold was adopted to eliminate false-negative predictions for high-rise reinforced concrete buildings, consistent with life-safety objectives. This performance gradient is physically consistent with the characteristics of the training data: high-rise reinforced concrete frames are more responsive to the spectral acceleration–related features (SDS, Year × PGA) captured by the model, whereas low-rise structures tend to fail due to unrecorded material deficiencies (e.g., poor cement quality) rather than geometric or configuration-related design shortcomings.

Achieving zero false negatives in the high-rise category is of paramount importance for life-safety screening. Considering the fact that even the failure of one multi-story building results in a disproportionately large number of fatalities compared to low-rise buildings, the full sensitivity of the model in the high-rise buildings is an indication of its reliability as a safety-based screening tool. In the test sample of 89 high-rise buildings, there were 65 Unsafe and 24 Safe buildings. The model was able to detect all 65 unsafe high-rise buildings (FN = 0), but it flagged only 22 out of 24 safe buildings.

**Table 7:** High-rise analysis.

Building Category	Storeys	Count	Accuracy	Recall (safety)
High-Rise	6+	89	0.753	1.0000 (100%)
Mid-Rise	3–5	284	0.644	0.9508 (95.1%)
Low-Rise	1–2	1086	0.653	0.8380 (83.8%)
Total	All	1459	0.657	0.8890 (88.9%)

### Stratified error analysis by construction era

In addition to validating the physical consistency of the framework further, stratified analysis of false negatives based on the construction period corresponding to significant upgrades to the Turkish Seismic Design Code was carried out. As presented in Table 8, it becomes clear that False Negative Rate (FNR), which represents the percentage of unsafe buildings not detected by the model, exhibits a strictly monotonic decline with the increasing level of modernization of buildings.

The highest value of the FNR achieved by the framework corresponds to the least modern category of buildings (Pre-1975), amounting to 22.2%, whereas the lowest FNR is observed for the most modern buildings (Post-2007) and equals 9.4%. It is obvious that this trend is consistent with physics; older buildings are likely to have unaccounted for stochastic factors such as deterioration of materials, hidden structural changes, and inadequate detailing, which cause noise in the macro-parameter space. Modern buildings, which were presumably constructed under strict supervision of the seismic codes and laws, exhibit damage states related to macro-seismic demand parameters (SDS, PGA) and geometrical properties (Storeys).

**Table 8:** Stratified false negative rate (FNR) by construction era.

Construction era	Corresponding seismic code context	False negative rate (FNR)
Pre-1975	Pre-modern regulations	22.20%
1975–1998	1975 Seismic design code	12.00%
1998–2007	1998 Seismic design code	10.70%
Post-2007	2007 & 2018 seismic design codes	9.40%

### Robustness and noise analysis

In order to evaluate the robustness of the proposed predictive model to realistic environmental disturbances, including surveyor error or GPS noise, the addition of independent random noise experiment was run. Random noise was continuously added to each input feature (SDS_DD2_IDW, PGA_DD2_IDW, IDW_6, STOREYS, YEAR_OF_CONST), the intensity of which was proportional to the observed range of values of each feature at different noise levels. Recalculations for interaction terms (STOREYS × YEAR, YEAR × PGA) were made, taking into account the noise added to the base features to ensure internal consistency. As is seen from Table 9, the model shows impressive stability, sustaining 86.2% accuracy even at the 5% perturbation level of input variables.

At the 10% noise level, Recall shifts relative to lower noise levels, behaving differently from Accuracy and Consistency. This result is explained by conservative uncertainty management and does not reflect an emergent fail-safe mechanism. Input perturbations shift borderline predicted probabilities toward the Unsafe class, since the asymmetric class weights and lowered decision threshold (0.40) cause ambiguous cases to be conservatively flagged, increasing false positives.

**Table 9:** Robustness (noise) analysis.

Noise level	Prediction consistency	Recall under noise
1% (Minor error)	91.8%	91.2%
5% (Moderate error)	86.2%	88.0%
10% (High error)	84.5%	90.1%

These noise levels were selected to approximate realistic field survey errors, GPS inaccuracies, and manual data-entry uncertainties observed in post-disaster rapid assessment campaigns.

### Leave-one-district-out spatial evaluation

The standard approach of randomized cross-validation may end up inflating the performance of the model because of spatial autocorrelation. For a rigorous evaluation of the robustness of the framework in terms of generalizability and vulnerability to domain shift, a novel approach to spatial validation known as leave-one-district-out was used. The ensemble model was trained from scratch based on data obtained from one district only, and then evaluated using the other district.

*Transfer Case 1 (Train: Nurdağı to Test: Islahiye)*:

The model trained on Nurdağı (49.6% unsafe rate) was tested on Islahiye (28.5% unsafe rate). The model achieved a Recall of 87.1% (identifying 1176 of 1350 unsafe structures) and an Accuracy of 45.6%.

*Transfer Case 2 (Train: Islahiye to Test: Nurdağı)*:

As shown in Table 10, when trained on Islahiye and tested on Nurdağı, the model achieved an exceptionally high recall of 96.7% (1,223 of 1,265), but accuracy dropped to 54.8% .

**Table 10:** Leave-one-district-out spatial transfer evaluation metrics.

Transfer scenario	Test set size (*n*)	Unsafe base rate	TP	FN	TN	FP	Recall	AUC	Accuracy
Case 1: Train Nurdağı, Test Islahiye	4744	28.50%	1176	174	985	2409	87.10%	0.683	45.60%
Case 2: Train Islahiye, Test Nurdağı	2548	49.60%	1223	42	174	1109	96.70%	0.726	54.80%

Note: TP = True Positive, FN = False Negative [Missed Risk], TN = True Negative, FP = False Positive [False Alarm]

While it is evident that the framework was successful in avoiding the potentially harmful false negatives, further examination of the confusion matrices shows that there was an increase in False Positives due to uncalibrated spatial transfer. More specifically, Transfer Case 2 showed 1109 False Positives, representing about 91% of the Nurdağı test dataset being classified as Unsafe.

The cause of this decrease in specificity lies in the very high skewness of the distribution shift. When one takes into account both the cost-sensitive parameter used in the framework (unsafe_w = 3.45) as well as the decreased threshold value (0.40), and considers the 28.5% damage rate among the population, the model decides to act cautiously when faced with a new environment of significantly different ground movements, along with an increased 49.6% damage rate. As such, in relation to the transfer of this decision-making model to a new environment, its predictions become Unsafe most of the time.

Overall, the conducted experiment proves the existence of one notable drawback related to data-driven frameworks in the sphere of RVS: while such systems can demonstrate robust generalization regarding building vulnerabilities, their application to new geographic areas with a very different prior damage probability distribution results in a lack of practical applicability.

## Discussion

### The accuracy-recall trade-off

As shown in Table 6, the proposed framework has an Accuracy of 65.73% with a Recall of 88.91%. In general machine learning tasks, an accuracy of 65% would be considered moderate. However, in the context of seismic risk mitigation, this accuracy and recall level is a necessary calibration. The cost of error in earthquake engineering is highly asymmetric. The cost of a False Positive (Type I error), where a safe building is labeled as risky, is a financial and logistical cost, where the municipality needs to send an engineer for a detailed inspection. On the other hand, the cost of a False Negative (Type II error), where a risky building is labeled as safe, is a catastrophic cost, where there could be loss of life in the next earthquake. By setting the decision threshold to 0.40 (as explained in Sect.  3.5), we have made it clear that we are giving a higher priority to the minimization of Type II errors. Although this strategy tends to increase the rate of False Positives, it also creates a strong ‘safety buffer.’ Specifically, out of the total number of 1,459 buildings in the testing set, 552 (37.8%) would be predicted as Safe, whereas 907 (62.2%) would need to be inspected. Out of 552 predicted as safe, 494 buildings (89.5%) were correctly predicted as safe, whereas 58 (10.5%) were wrongly predicted as safe. By extrapolation to the whole data set of 7,292 buildings, there would be 2,759 buildings predicted as safe, and 4,533 buildings requiring inspection, which is an estimated 37.8% decrease in effort compared to universal inspection. Based on an average inspection rate of 200 buildings per day (10 teams of engineers × 20 buildings per engineer team), 22.7 days would be required to investigate the pool of buildings, compared to 36.5 days needed by the universal method.

### Interpretability via SHAP

**Fig. 12:**
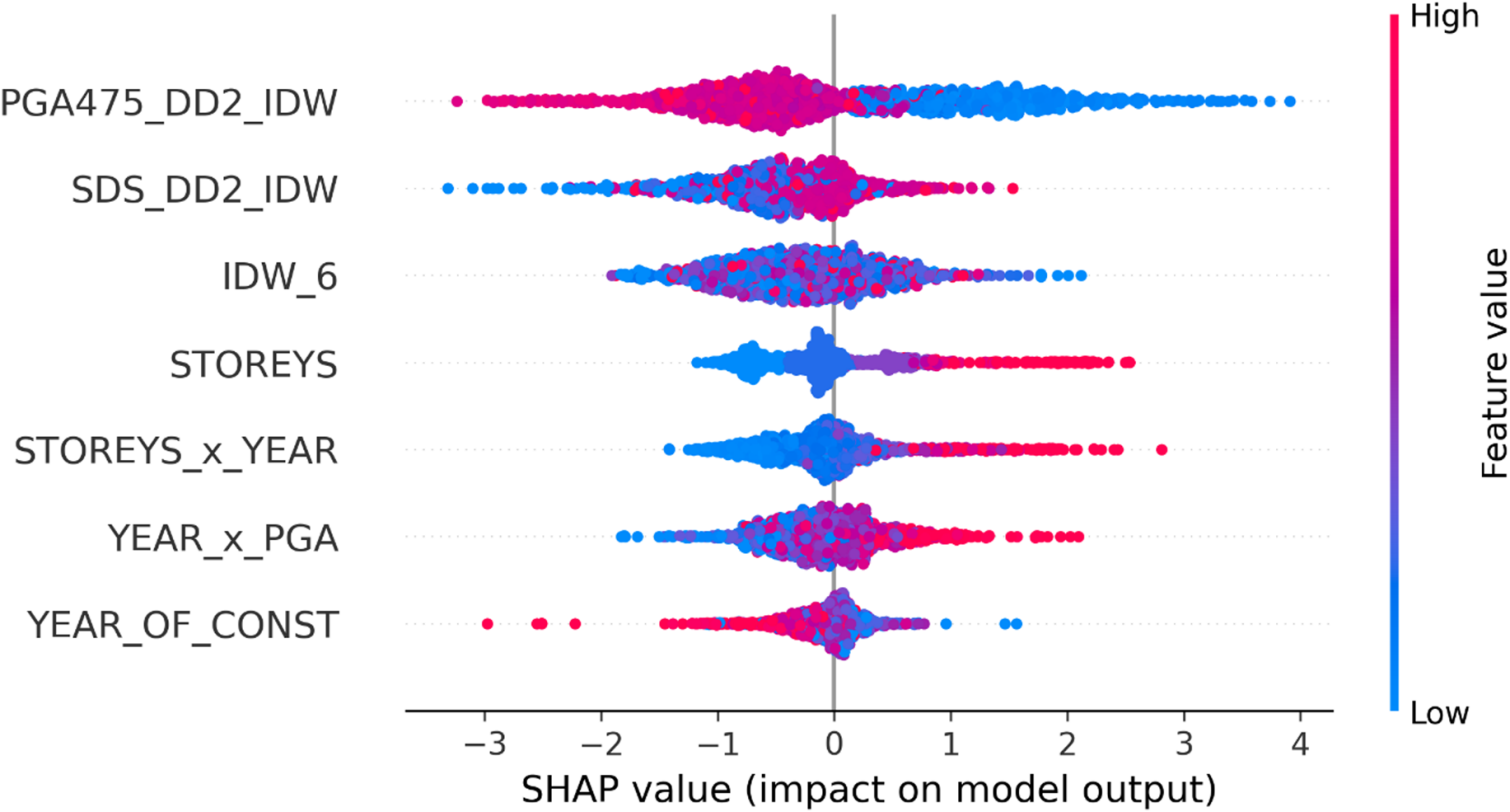
SHAP summary plot.

Figure 12 shows the summary plot for SHAP, which gives a transparent view of the logic used by the model for its decision-making. The feature hierarchy also validates the effectiveness of the engineering-informed approach.

The feature YEAR × PGA, representing the interaction between Building Age and Seismic Demand, is used as a significant feature in the model. As seen in the plot, the high feature values (red dots), representing older buildings under high PGA demand, are the dominant group located on the positive side of the x-axis. From the STOREYS feature, it is evident that there is a clear trend in which, when values are high (as depicted by red points, which indicate high-rise buildings), SHAP values will always be positive, contributing to risk, and when values are low (as depicted by blue points), they contribute to safety. There is a clear pattern of the SDS_DD2_IDW spectral acceleration parameter wherein increasing levels of seismic intensities (red color) move the probabilities to the ‘Unsafe’ class, consistent with the principles of earthquake engineering.

### Comparison with heuristics

In the case of seismic risk mitigation in Turkey, the “Pre-1998 Rule” is the commonly used heuristic, whereby it categorically classifies buildings constructed before the 1998 Seismic Code as “high risk.” This is a very sensitive strategy but low in specificity, since it will classify many of the strong, low-rise buildings built prior to the 1998 codes as being of high risk.

The proposed ensemble model is evidently better than this baseline in its discriminative ability. The Storeys × Year term can be used to differentiate among the different types of buildings: Old and Vulnerable (mid- to high-rise buildings with poor detail quality) and Old but Stable (low-rise masonry or rigid frames), with the help of this model. The machine learning model also reduces the False Positive Rate of the age-based model significantly, as it assists the municipal authorities in redistributing the engineering resources allocation in the city to include the buildings that are classified as critical within the high-rise and high PGA zones, instead of relying on the buildings that are old but stable. The framework is particularly suited for reinforced concrete building inventories common in seismically active urban regions.

Table 11 provides a quantitative comparison between the Pre-1998 heuristic and the proposed ensemble on the independent test set. While the heuristic achieves higher specificity (61.5% vs. 52.8%), it misses 289 of 523 unsafe buildings — nearly five times more than the ensemble (58 missed). The ensemble’s recall advantage of 44.2% points translates directly into avoided casualties: every missed unsafe building represents a structure cleared for occupation that has a compromised structural system.

**Table 11:** Quantitative comparison of pre-1998 heuristic vs. safety-first ensemble on the independent test set (*n* = 1459).

Metric	Pre-1998 heuristic	Ensemble (θ = 0.40)
Rule	Flag all YEAR < 1998	Soft-voting ensemble, cost-sensitive
Recall (unsafe)	44.74%	88.91%
Specificity (safe)	61.54%	52.78%
Accuracy	55.52%	65.73%
Buildings flagged	594 (40.7%)	907 (62.2%)
Missed unsafe buildings	289	58

The heuristic flags 313 fewer buildings for inspection (lower operational burden), but at the cost of missing 231 additional unsafe buildings. The ensemble achieves this by incorporating seismic demand, building height, and demand-capacity interaction features that the binary age-rule cannot represent. Notably, the heuristic completely fails to differentiate between a 10-storey pre-1998 building in a high-PGA zone; the most casualty-critical combination; and a 1-storey pre-1998 building in a low-demand area. The ensemble’s STOREYS×YEAR interaction term makes exactly this distinction.

## Conclusion

In order to include the effects of changing seismic design codes, the buildings were linked with the currently used seismic code at the time of design. The assumption was that the projects of the structural design were made ready about one year before the year of construction was recorded. The seismic code that was in effect during that design year was therefore adopted as the governing code of each building.

The framework of Engineering-informed Ensemble Learning, which is the outcome of the study, holds the promise of the quick screening of the seismic risks of RC buildings. Inserting XGBoost, LightGBM, and CatBoost into the loop of optimization in the HPC environment, we have developed a screening tool that prioritizes life safety over purely statistical objectives.

Key results of the study are as follows:

- The model achieved a Recall score of 88.91% on an unseen test set, implying that the model was highly sensitive to structural defects. By fine-tuning the decision threshold to 0.40, the framework was able to effectively minimize catastrophic false negatives.
- With a 100% detection rate being achieved for high-rise buildings (6 + storeys), the majority of earthquake-related building collapse casualties come from multiple-storey building collapse events.
- The feature engineering and SHAP analysis indicated that the model learned some hidden seismic interactions, such as the cumulative risk of aging frames due to increased spectral acceleration demands (Year × PGA), rather than relying on data artifacts.
- Under noise levels below 10%, the network exhibits conservative uncertainty behavior in which the asymmetry between the two classes’ weights along with the reduction in the threshold leads to the prediction bias toward the Unsafe class. Consequently, recall increases at the cost of an increase in false positives. This is a design feature of the safety-first approach adopted here, not an emergent result of the neural network design. The suggested framework can serve as a reliable Tier-1 screening model that reduces the amount of inspection load required by around 37.8% while recognizing about 89% of unsafe structures for engineering evaluation.
- The limitation of the presented approach is its use of code-based seismic hazard maps (DD-2), instead of event-specific ShakeMaps, as a proxy of seismic demand. Though this approach provides the benefit of immediate availability, without waiting for instrumental data collection and assimilation, it cannot account for the true spatial variation in shaking intensity due to the complex geophysical processes such as rupture directivity and near-fault pulses that took place during the 2023 Kahramanmaraş earthquakes. Future developments of the present framework would consider using a hybrid approach where a screening with hazard maps, which are immediately available after the mainshock event (hour zero), is followed by updates of the machine learning risk predictions when high-fidelity ShakeMaps become available.

Future research should address three principal directions. First, structural information enrichment: a key limitation of the current framework is its reliance on easily obtainable but limited building-level attributes. Recent advances demonstrate that hidden structural information (including the spatial arrangement of shear walls and material strength grades) can be reconstructed at the urban scale from limited observable data^26,27^, while deep learning-based methods can predict floor-level inter-storey drift ratios and peak floor accelerations from limited building parameters and ground motion inputs^28,29^, including under mainshock–aftershock sequences directly relevant to the 2023 Kahramanmaş doublet^30^. A natural evolution of the present framework would fuse such enriched vulnerability features and predicted engineering demand parameters with the data-driven ensemble classifier for finer damage state identification beyond binary classification.

Second, cross-domain adaptation: the framework is trained and validated exclusively on the 2023 Kahramanmaş dataset from the Gaziantep region. Deploying it in a different seismic region would encounter domain shift from differences in building inventory, construction practice, seismic demand characteristics, and damage labelling protocols. Conventional transfer learning may be inadequate when target-domain labelled data are scarce — as is typical in the immediate aftermath of a new earthquake. A multi-fidelity data meta-learning approach^31^ offers a principled solution, enabling rapid adaptation to new building types and seismic scenarios with minimal target-domain data, and providing a credible methodological route for extending this framework to other earthquakes and jurisdictions. Third, computer vision techniques to aid the automatic extraction of visual building parameters would further reduce reliance on manual data entry in future deployments.

## Data Availability

The building-level damage assessment data used in this study were **officially purchased** from the Ministry of Environment, Urbanization and Climate Change of Türkiye following the 6 February 2023 Kahramanmaraş earthquakes and were provided to the authors in Excel format. Due to licensing and data use agreements, the raw datasets cannot be made publicly available. However, processed and anonymized data products derived from the original dataset, including aggregated damage classifications and spatial analysis results, can be made available from the corresponding author upon reasonable request.
